# Trends in mortality after a sepsis hospitalization: a nationwide prospective registry study from 2008 to 2021

**DOI:** 10.1007/s15010-023-02082-z

**Published:** 2023-08-12

**Authors:** Nina Vibeche Skei, Tom Ivar Lund Nilsen, Randi Marie Mohus, Hallie C. Prescott, Stian Lydersen, Erik Solligård, Jan Kristian Damås, Lise Tuset Gustad

**Affiliations:** 1 Department of Anesthesia and Intensive Care, Nord-Trondelag Hospital Trust, Levanger, Norway; 2 Department of Circulation and Medical Imaging, Mid Norway Sepsis Research Center, Norwegian University of Science and Technology (NTNU), Trondheim, Norway; 3 Department of Public Health and Nursing, Norwegian University of Science and Technology (NTNU), Trondheim, Norway; 4 Clinic of Anesthesia and Intensive Care, St. Olav’s University Hospital, Trondheim, Norway; 5 Department of Internal Medicine, University of Michigan, Ann Arbor, MI USA; 6 VA Center for Clinical Management Research, Ann Arbor, MI USA; 7 Department of Mental Health, Faculty of Medicine and Health Sciences, Regional Centre for Child and Youth Mental Health and Child Welfare, Norwegian University of Science and Technology, (NTNU), Trondheim, Norway; 8 Centre of Molecular Inflammation Research, Institute for Clinical and Molecular Medicine, Norwegian University of Science and Technology (NTNU), Trondheim, Norway; 9 Department of Infectious Diseases, St. Olav’s University Hospital, Trondheim, Norway; 10 Faculty of Nursing and Health Sciences, Nord University, Levanger, Norway; 11 Department of Medicine and Rehabilitation, Levanger Hospital, Nord-Trøndelag Hospital Trust, Levanger, Norway

**Keywords:** Mortality, Sepsis, COVID-19, Intensive care

## Abstract

**Background:**

Few studies have reported on mortality beyond one year after sepsis. We aim to describe trends in short- and long-term mortality among patients admitted with sepsis, and to describe the association between clinical characteristics and mortality for improved monitoring, treatment and prognosis.

**Methods:**

Patients ≥ 18 years admitted to all Norwegian hospitals (2008–2021) with a first sepsis episode were identified using Norwegian Patient Registry and International Classification of Diseases 10th Revision codes. Sepsis was classified as implicit (known infection site plus organ dysfunction), explicit (unknown infection site), or COVID-19-related sepsis. The outcome was all-cause mortality. We describe age-standardized 30-day, 90-day, 1-, 5- and 10-year mortality for each admission year and estimated the annual percentage change with 95% confidence interval (CI). The association between clinical characteristics and all-cause mortality is reported as hazard ratios (HRs) adjusted for age, sex and calendar year in Cox regression.

**Results:**

The study included 222,832 patients, of whom 127,059 (57.1%) had implicit, 92,928 (41.7%) had explicit, and 2,845 (1.3%) had COVID-19-related sepsis (data from 2020 and 2021). Trends in overall age-standardized 30-day, 90-day, 1- and 5-year mortality decreased by 0.29 (95% CI − 0.39 to − 0.19), 0.43 (95% CI − 0.56 to − 0.29), 0.61 (95% CI − 0.73 to − 0.49) and 0.66 (95% CI − 0.84 to − 0.48) percent per year, respectively. The decrease was observed for all infections sites but was largest among patients with respiratory tract infections. Implicit, explicit and COVID-19-related sepsis had largely similar overall mortality, with explicit sepsis having an adjusted HR of 0.980 (95% CI 0.969 to 0.991) and COVID-19-related sepsis an adjusted HR of 0.916 (95% CI 0.836 to 1.003) compared to implicit sepsis. Patients with respiratory tract infections have somewhat higher mortality than those with other infection sites. Number of comorbidities was positively associated with mortality, but mortality varied considerably between different comorbidities. Similarly, number of acute organ dysfunctions was strongly associated with mortality, whereas the risk varied for each type of organ dysfunction.

**Conclusion:**

Overall mortality has declined over the past 14 years among patients with a first sepsis admission. Comorbidity, site of infection, and acute organ dysfunction are patient characteristics that are associated with mortality. This could inform health care workers and raise the awareness toward subgroups of patients that needs particular attention to improve long-term mortality.

**Supplementary Information:**

The online version contains supplementary material available at 10.1007/s15010-023-02082-z.

## Background

Sepsis occurs when a dysregulated immune response to infection leads to tissue damage and organ dysfunction [1]. This heterogeneous syndrome is associated with a high risk of death and is estimated to cause 20% of all global deaths [2]. While mortality up to 1 year and declining case fatality trends are well documented among sepsis patients [3–7], two recent studies report no change in short- and long-term mortality trends in sepsis patients admitted to intensive care units (ICU) with sepsis [8, 9]. Information on trends in long-term mortality beyond one-year among all hospitalized sepsis patients, including those admitted to the wards, is limited [10, 11]. Further, to commission appropriate health services, contemporary trends are needed to meet the increased use of healtcare [12].

Identifying the site of infection is one of the keys in the management of sepsis [13]. Respiratory tract infections being the most common site, followed by abdomen, bloodstream, and genitourinary infections [12–14]. During the recent pandemic, an unprecedented number of patients were admitted with respiratory tract infection due to the novel SARS-CoV-2 virus and developed sepsis [14–16]. Thus, the pathogen and infection site in these cases were known, limited targeted treatment could be offered [17], and the long-term outcomes beyond 1 year of COVID-19-related sepsis is limited.

In-hospital mortality trends based on the site of infection in sepsis patients are declining for all sites [18]; however, little is known about mortality trends beyond hospital discharge. Moreover, there are conflicting results regarding the prognostic impact of infection sites on long-term mortality, with two studies conducted on ICU patients estimating that all infection sites had higher long-term mortality than respiratory tract infections [19, 20], while others reported the opposite [4, 21]. It is well known that worsen and new comorbidity contributes to higher mortality in sepsis patients [22]. Interestingly, a recent study found that more than 20% of the patients who survived sepsis had a late death that not could be explained by health status before sepsis and suggests that the sepsis itself contributes to poor long-term outcomes [23]. However, little is known about the impact of infection sites on long-term mortality in hospitalized sepsis patients beyond ICU cohorts and short-term follow-up.

Sepsis patients develop acute organ dysfunction, and the organs most often affected are kidneys, liver, lungs, cardiovascular and hematological system [24]. An increasing number of acute organ dysfunctions has been associated with an increased risk of early death in sepsis survivors [4]. A two-year follow-up multicenter study of sepsis patients found that neurologic dysfunction had the strongest adverse impact on long-term mortality, whereas other types of organ dysfunctions had a relatively modest impact [25]. Studies estimating the association between acute organ dysfunction and long-term mortality are few and restricted to specific sepsis diagnosis or have only included patients in ICUs or emergency departments [25, 26]. These studies may not fully capture the broader population of hospitalized sepsis patients or those who acquire sepsis during the hospital stay for other medical conditions [27].

In this nationwide study, we describe temporal trends in short- (30-day) and long-term (90-day, 1-, 5-, and 10-year) mortality over the past 14 years, including the recent COVID-19 pandemic, among patients admitted with a first-time sepsis, both overall and for subgroups of sepsis patients. Lastly, we investigate clinical characteristics associated with long-term mortality.

## Methods

### Study design and population

We conducted a prospective nationwide registry study, using data on all patients $\ge$ 18 years with ICD-10 discharge codes for sepsis admitted to Norwegian hospitals in the period January 1, 2008, through.

December 31, 2021. The data were provided by The Norwegian Patient Registry on an individual level using the personal identification number [28]. Reporting to the Norwegian Patient Registry is mandatory and NPR data is shown to have high level of completeness [28]. The Norwegian Patient Registry data were also linked to the Norwegian Intensive Registry [29], which covers all intensive care admissions since May 1, 2014.

**Fig. 1:**
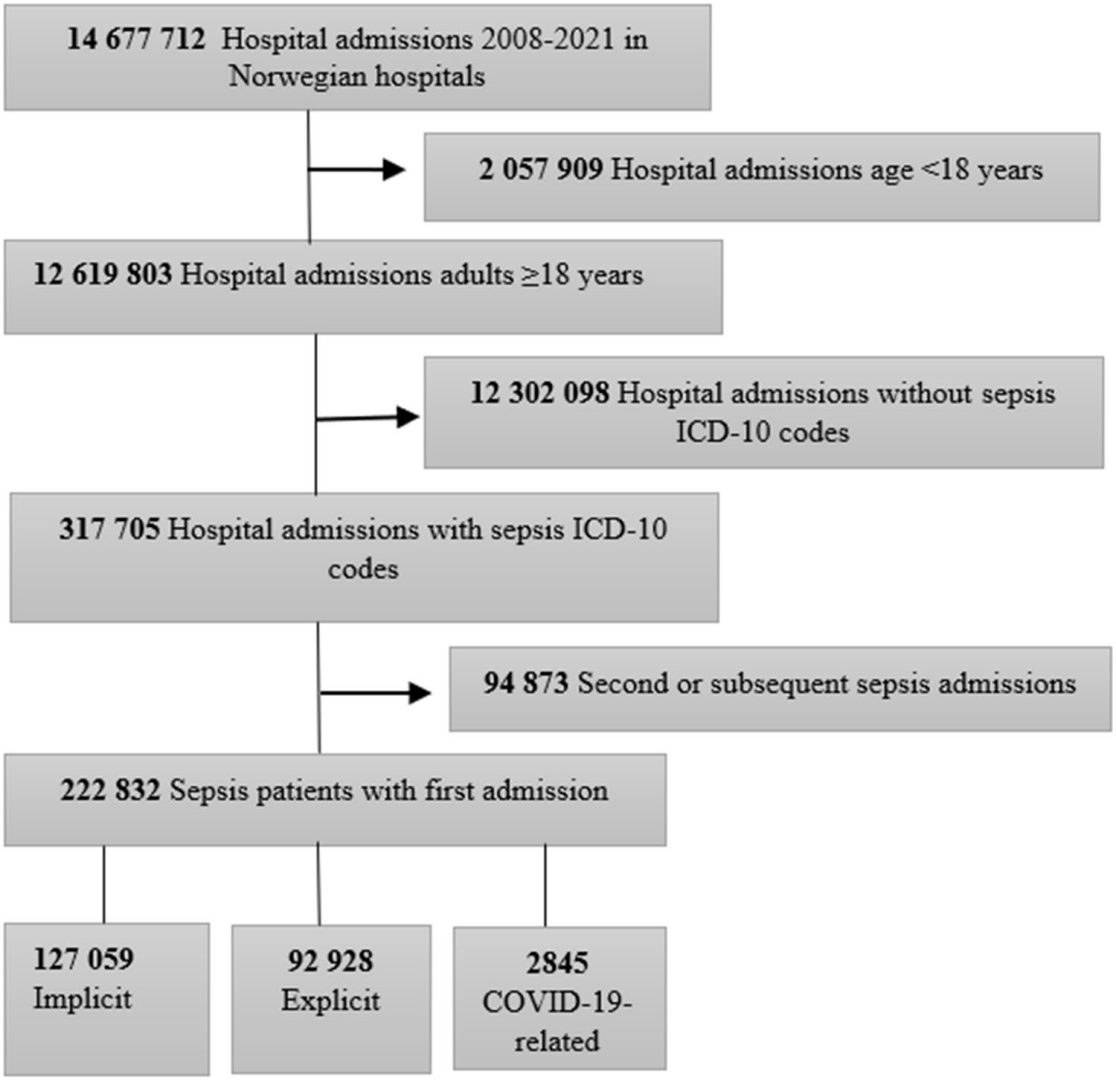
Flowchart of the selection process

We included the first admissions for sepsis during the period 2008 through 2021. We used the Sepsis-3 definition (2016) to define sepsis (presence of acute infection and acute new organ dysfunction)^1^.We followed the approach used by Rudd et al. and extracted codes for implicit and explicit sepsis [2]. Implicit sepsis cases were those recognized with an ICD-10 discharge code for infection plus acute organ dysfunction, while explicit sepsis cases were those recognized with an specific sepsis ICD-10 discharge code. COVID-19-related sepsis was included based on the presence of a discharge code for COVID-19 (U07.1, U07.2) and ≥ one organ dysfunction code and/or explicit code. We used this strategy in the primary and up to 20 secondary co-existing ICD-10 discharge codes. We report estimates for all sepsis cases combined (implicit, explicit and COVID-19-related sepsis) and for each subgroup. The patient was classified as an implicit sepsis case only if the patient did not meet the criteria for an explicit sepsis or COVID-19-related sepsis, similar to the code extraction strategy of Rudd et al. (2020). In addition, we categorized infection, comorbidities and acute organ dysfunctions by ICD-10 discharge codes. Acute neurological dysfunction was not characterized as a single acute organ dysfunction but included in the category of other acute dysfunctions. ICD-10 discharge codes for selected comorbidities were based on diagnostic groups [30]. We provide an overview of the ICD-10 codes in the Supplemental Files, Supplemental Methods. Among 12,619,803 adult hospital admissions ≥ 18 years, 317,705 (2.5%) patients met the criteria for sepsis, and of these 222,832 were hospitalized with a first episode of sepsis in the study period (Fig. 1).

### Outcomes

The primary outcome was all-cause mortality obtained from a linkage between the NPR records and The Norwegian Cause of Death Registry, covering all Norwegian citizens [31]. Mortality was calculated as the proportion of deaths of any cause among those admitted with sepsis during a specific year. Patients were followed from January 1, 2008, to December 31, 2021, and censored at their date of death and last death date was ascertained December 31, 2021.

### Statistical analysis

Descriptive characteristics of the population are presented as frequencies with percentages, means with standard deviations, and medians as appropriate and shown for all sepsis patients, as well as stratified according to sepsis, and COVID-19-related sepsis. For each calendar year, we estimated 30-day, 90-day, 1-, 5-, and 10-year mortality by calculating the proportions of deaths from all causes, divided by the number of first sepsis admissions. The estimated mortality proportion was standardized according to age groups (18–29, 30–39, 40–49, 50–59, 60–69, ≥ 80 years) using the age distribution in 2009 as the base. Temporal trends in age-standardized mortality were estimated from least-squares linear regression across calendar years (2009–2021) and weighted by the inverse variance of the mortality proportion for all patients with a first sepsis epidose [32]. The year 2008 was excluded from trend analyses due to the increased likelihood of including recurrent and more severe sepsis episodes in the first year of observation. Similar analyses were conducted for subgroups of sepsis patients according to diagnosis, infections site, comorbidities. Analyses of patients receiving intensive care treatment or who were admitted to the ward was restricted to the period May 1, 2014, to December 31, 2021, since earlier information was not available.

The association between clinical characteristics (i.e., comorbidity, infection site, and acute organ dysfunction) and mortality were estimated by Cox regression with time to death as a dependent variable. First, we included each characteristic separately (crude). Thereafter we adjusted for sex age, the years 2009 to 2019 as a continuous covariate, and the years 2008, 2020 and 2021 as separate indicator variables to allow for deviations from a linear association in the first year of observation and during the pandemic years. The patient characteristics were type of sepsis diagnosis (i.e., implicit, explicit, and COVID-19-related sepsis), type and number of comorbidities, infection site, number and type of acute organ dysfunction, and intensive care treatment. Comorbidities, infection sites, and acute organ dysfunctions were analyzed as categorical variables, using the most frequent category as a reference. The categories were mutually exclusive, and the analyses were therefore conducted on a restricted sample of patients with none or only one comorbidity, infection site, or acute organ dysfunction, respectively.

We report crude and adjusted hazard ratios (HRs) with 95% CIs. In the survival analyses the patients came at risk at the date of first admission and were censored at the death date or last day of follow-up (December 31, 2021). In the analysis assessing mortality in ICU patients compared to ward patients, both the ward and ICU patients entered the study after May 1, 2014, since earlier information was not available for the ICU patients. The proportional hazards assumption of the Cox model was examined by visual inspection of log–log plots.

Sensitivity analysis was conducted to account for the late entry of COVID-19-related sepsis patients. We used a similar Cox model as described above, but with follow-up time starting from February 27, 2020, for all patients with implicit, explicit, and COVID-19-related sepsis. The entry date corresponds with the first confirmed hospitalized COVID-19 case in Norway. Since many patients have more than one infection site, comorbidity and acute organ dysfunction, we also analyzed separate binary variables for each infection site, comorbidities and acute organ dysfunction (i.e., 0 = No, 1 = Yes).

All analyses were conducted using STATA version 16.1 (Stata Corp).

### Ethics

The study was approved by the Regional Committee for Medical and Health Research Ethics (REK) in Eastern Norway (2019/ 42,772) and the Data Access Committee in Nord-Trøndelag Hospital Trust (2021/184). In accordance with the approval from the REK and the Norwegian law on medical research, the project did not require written patient consent. This work was analyzed on TSD (Service for Sensitive Data) facilities owned by the University of Oslo, operated, and developed by the TSD service group at the University of Oslo, IT Department (USIT). TSD is designed for storing and post-processing sensitive data in compliance with the Norwegian "Personal Data Act" and "Health Research Act."

## Results

### Patient characteristics

The patient characteristics at first admission with sepsis and subgroups of sepsis are shown in Table 1. Among patients with first hospitalization for sepsis, the proportion of men was 54.1%, variating between 52.7% (implicit group), 55.6% (explicit group), and 65.5% (COVID-19-related sepsis group). Chronic heart and vascular disease was the most frequent comorbidity in 44.9% of all sepsis patients, with 48.2% in the implicit group, 41.0% in the explicit group, and 24.7% in the COVID-19-related sepsis group. Readmission within 30 days after the first hospitalization for all sepsis patients was 24.9% and occurred in 24.3% of the patients with implicit sepsis, in 25.9% of the patients with explicit sepsis, and in 16.7% of the patients with COVID-19-related sepsis. Overall the respiratory tract was the most common infection site with 36.8% and diagnosed in 50.2% of the implicit sepsis patients and in 91.1% of the COVID-19-related sepsis patients. Overall 8.5% of the sepsis patients were admitted to the ICU, and in the subgroups 8.7% of implicit sepsis patients, 7.9% of the explicit sepsis patients (data from 2014 to 2021), and 11.1% of those with COVID-19-related sepsis needed ICU treatment. (data from 2020 to 2021).

**Table 1:** Characteristic of the study population with sepsis (2008–2021), including subgroups

	Sepsis^a^	Subgroups of sepsis		
		Implicit^b^	Explicit^c^	COVID-19-related^d^
Characteristics				
First admission, *n* (% of all)	222,832 (100)	127,059 (57.0)	92,928 (41.7)	2845 (1.3)
Male, *n* (%)	120,442 (54.1)	66,929 (52.7)	51,651 (55.6)	1862 (65.5)
Mean age, years (SD)	71.1 (16.6)	73.0 (15.7)	68.9 (17.5)	61.4 (16.1)
Comorbidities, *n* (%)				
Heart and vascular	100,062 (44.9)	61,251 (48.2)	38,109 (41.0)	702 (24.7)
Cancer	39,368 (17.7)	17,270 (13.6)	21,973 (23.6)	125 (4.4)
Lung	36,165 (16.2)	26,993 (21.2)	8866 (9.5)	306 (10.8)
Renal	8949 (4.0)	5830 (4.6)	3043 (3.3)	76 (2.7)
Diabetes	24,416 (10.9)	13,682 (10.8)	10,348 (11.1)	386 (13.6)
Dementia	8100 (3.6)	4561 (3.6)	3507 (3.8)	32 (1.1)
Immune	3140 (1.4)	1640 (1.3)	1451 (1 0.6)	49 (1.7)
Liver	994 (0.5)	564 (0.4)	427 (0.5)	≤ 5
Number of comorbidities, *n* (%)				
0	68,450 (30.7)	36,185 (28.5)	30,684 (33.0)	1581(55.6)
1	98,803 (44.3)	56,884 (44.7)	41,050 (44.2)	909 (32.0)
2	45,352 (20.4)	27,768 (21.9)	17,284 (18.6)	300 (10.5)
≥ 3	10,227 (4.6)	6262 (4.9)	3910 (4.2)	55 (1.9)
Site of infection, *n* (%)				
Respiratory	81,881 (36.8)	63,724 (50.2)	15,566 (16.8)	2591 (91.1)
Genitourinary	44,782 (20.1)	28,838 (22.7)	15,862 (17.1)	82 (2.9)
Skin and soft tissue	8265 (3.7)	3578 (2.8)	4682 (5.0)	5 (0.2)
Gastrointestinal	10,810 (4.8)	8356 (6.6)	2424 (2.6)	30 (1.1)
Intra-abdominal	12,340 (5.5)	5401 (4.3)	6917 (7.4)	22 (0.8)
Infections following a procedure	8290 (3.7)	4042 (3.2)	4235 (4.6)	13 (0.5)
Endocarditis/myocarditis	2530 (1.1)	1008 (0.8)	1514 (1.6)	8 (0.3)
Other^e^	43,085 (19.3)	24,463 (19.3)	18,434 (19.8)	188 (6.6)
Organ system with acute dysfunction, *n* (%)				
Respiratory	61,864 (27.8)	51,453 (40.5)	8012 (8.6)	2399 (84.3)
Circulatory	14,892 (6.7)	10,647 (8.4)	4177 (4.5)	68 (2.4)
Renal	67,242 (30.2)	54,295 (42.7)	12,514 (13.5)	433 (15.2)
Hepatic	3209 (1.4)	2178 (1.7)	1014 (1.1)	17 (0.6)
Coagulation	6471(2.9)	3858 (3.1)	2570 (2.8)	43 (1.5)
Other^f^	22,173 (10.0)	20,095 (15.8)	1928 (2.1)	150 (5.3)
Number of acute organ dysfunctions, *n* (%)				
1	133,808 (87.7)	113,998 (89.7)	17,339 (76.0)	2471 (86.9)
2	15,262 (10.0)	11,038 (8.7)	3955 (17.3)	269 (9.5)
3	2864 (1.9)	1693 (1.3)	1144 (5.0)	27 (0.9)
≥ 4	699 (0.5)	330 (0.3)	264 (1.6)	≤ 5
Number of hospital admissions for sepsis^g^, *n* (%)				
1	171,619 (77.0)	97,105 (76.4)	71,800 (77.3)	2714 (95.4)
2	33,221 (14.9)	19,339 (15.2)	13,757 (14.8)	125 (4.4)
3	10,129 (4.6)	5917 (4.7)	4208 (4.5)	≤ 5
4	4011 (1.8)	2363 (1.9)	1647 (1.8)	≤ 5
≥ 5	3852 (1.7)	2335 (1.8)	1516 (1.6)	≤ 5
Readmission^h^, *n* (%)	55,441 (24.9)	30,895 (24.3)	24,072 (25.9)	474 (16.7)
ICU treatment^j^, *n* (%)	10,602 (8.5)	6946 (8.7)	3341 (7.9)	315 (11.1)
In-hospital death, *n* (%)	30,276 (13.6)	16,273 (12.8)	13,751 (14.8)	352 (12.4)

*ICU* intensive care unit

^a^Sepsis = All first sepsis admissions in the period 2008–2021, including implicit, explicit and COVID-19-related sepsis (2020–2021)

^b^Implicit sepsis = ICD-10 code for infection in combination with a code for acute organ function, excluding those who had an explicit code at the same hospital admission

^c^Explicit sepsis = ICD-10 code for specific sepsis, including those who also had an implicit code at the same admission

^d^COVID-19-related sepsis = ICD-10 code for COVID-19 in combination with an acute organ dysfunction code and/or a specific sepsis code

^e^Other infections = Bone, obstetric, upper airway, central nervous system and unknown

^f^Other acute organ dysfunction = Acidosis, unspecific gangrene, central nervous system dysfunctions and Systemic Inflammatory Response Syndrome

^g^Number of hospital admissions = Calculated as new sepsis admission if admission with ICD-10 codes defining sepsis, regardless of time frame for the new sepsis admission

^h^Readmission = admission within 30 days after discharge regardless of cause

^j^Variable calculated from May 1, 2014

### Temporal trends in mortality

**Fig. 2:**
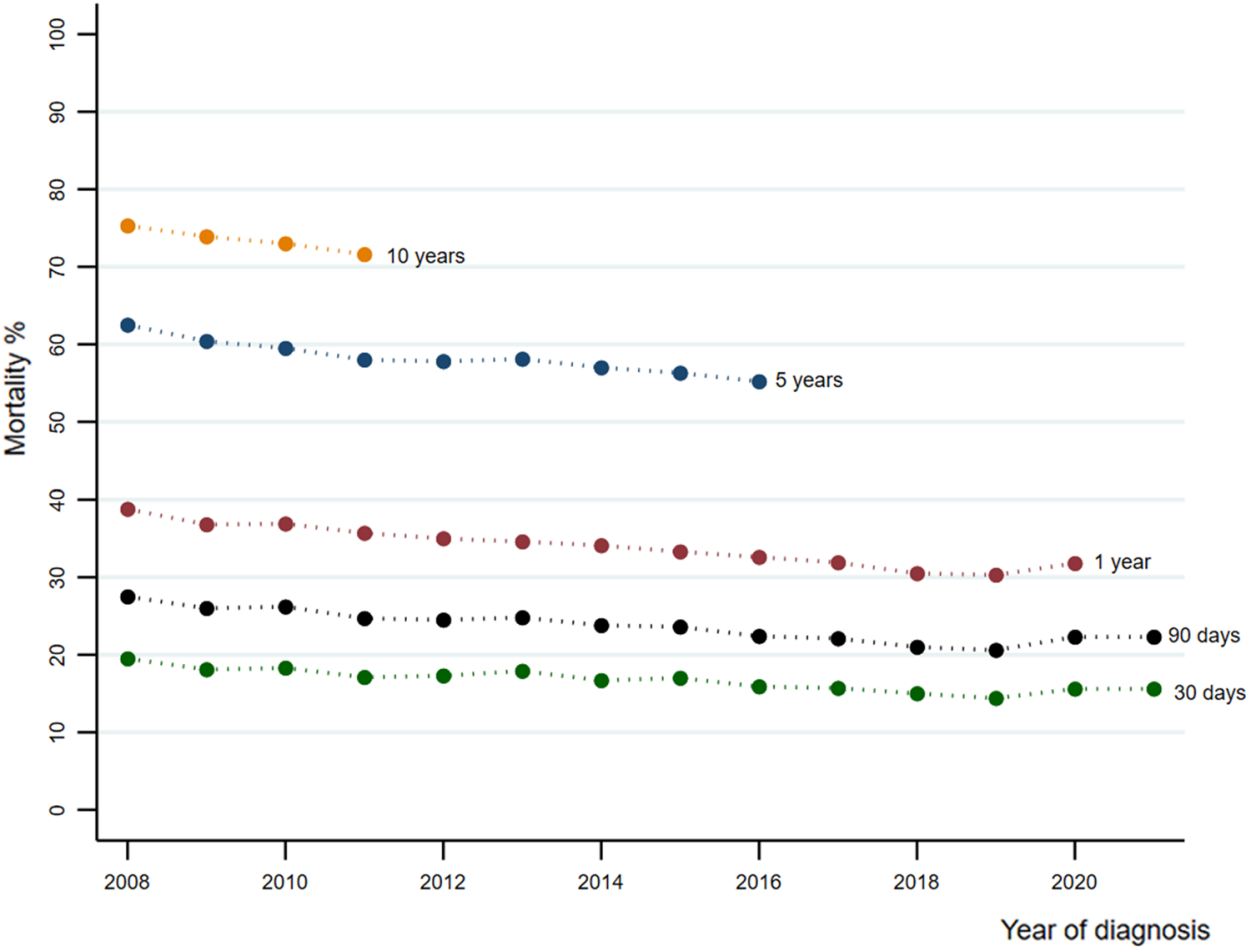
Age-standardized mortality at 30-day, 90-day, 1-, 5- and 10-year according to admission year for all patients hospitalized with a first sepsis

The 30-day age-standardized mortality for patients admitted with a first sepsis episode declined 0.29% (95% CI − 0.39 to − 0.19) per year from 18.2% (95% CI 17.6 to 18.8) to 15.9% (95% CI 15.4 to 16.5), while the 90-day declined 0.43% (95% CI − 0.56 to − 0.29) per year from 26.0% (95% CI 25.3 to 26.7) to 22.3% (95% CI 21.6 to 23.0). The 1-year age-standardized mortality declined 0.61% (95% CI − 0.73 to − 0.49) per year from 36.8% (95% CI 36.1 to 37.6) to 31.8% (95% CI 31.1 to 32.5), while the 5-year declined 0.61% (95% CI − 0.73 to − 0.49) from 60.4% (95% CI 59.7 to 61.1) to 55.2% (95% CI 54.5 to 55.9) and the 10-year age-standardized mortality declined 1.23% per year (95% CI − 2.91 to 0.63) from 73.4% (95% CI 72.8 to 73.9) to 71.0% (95% CI 70.4 to 71.6) (Fig. 2 and Table 2). Subgroup analysis for patients reciving intensive care was stable from 2014 and througout the study period, shown in Supplementary Files, Supplementary Fig. 1.

**Table 2:** Age-standardized percentage change per year in 30- and 90-day, 1- and 5-year mortality^a^ in overall and within different subgroups (2008–2021)

	*n*	30-day	90-day	1-year	5-year
Group					
All first sepsis patients	222,832	− 0.29 (− 0.39, − 0.19)	− 0.43 (− 0.56, − 0.29)	− 0.61 (− 0.73, − 0.49)	− 0.66 (− 0.84, − 0.48)
Implicit	127,059	− 0.31 (− 0.43, − 0.19)	− 0.43 (− 0.60, − 0.25)	0.68 (− 0.87, − 0.49)	− 1.01 (− 1.19, − 0.83)
Explicit	92,928	− 0.19 (− 0.31, − 0.07)	− 0.32 (− 0.46, − 0.18)	− 0.40 (− 0.52, − 0.29)	− 0.39 (− 0.66, − 0.12)
ICU^b^	10,602	− 0.20 (− 0.62, 0.22)	− 0.39 (− 0.87, 0.89)	− 0.72 (− 1.37, − 0.08)	NA
Ward	212,230	− 0.39 (− 0.51, − 0.27)	− 0.53 (− 0.68, − 0.37)	− 0.70 (− 0.84, − 0.57)	− 0.73 (− 0.92, − 0.54)
Infection site^c^					
Respiratory	66,368	− 0.38 (− 0.57, − 0.19)	− 0.58 (− 0.82, − 0.34)	− 0.84 (− 1.04, − 0.65)	− 1.02 (− 1.25, − 0.79)
Genitourinary	28,938	− 0.13 (− 0.25, 0.004)	− 0.30 (− 0.47, − 0.13)	− 0.51 (− 0.71, − 0.30)	− 0.71 (− 1.00, − 0.43)
Skin and soft tissue	3583	− 0.06 (− 0.27, 0.16)	− 0.21 (− 0.43, 0.03)	− 0.49 (− 0.78, − 0.20)	− 0.98 (− 1.84, − 0.12)
Gastrointestinal	8394	− 0.13 (− 0.29, 0.02)	− 0.21 (− 0.44, 0.01)	− 0.42 (− 0.74, − 0.10)	− 0.40 (− 1.13, 0.33)
Intra− abdominal	5437	− 0.27 (− 0.46, − 0.07)	− 0.52 (− 0.74, − 0.31)	− 0.55 (− 0.74, − 0.57)	− 0.41 (− 1.07, 0.25)
Infections following a procedure	4070	− 0.06 (− 0.26, 0.14)	− 0.30 (− 0.58, − 0.02)	− 0.48 (− 0.83, − 0.14)	− 0.81 (− 1.76, 0.14)
Endocarditis/ Myocarditis	1020	− 0.29 (− 0.75, 0.17)	− 0.47 (− 0.90, − 0.04)	− 0.63 (− 1.12, − 0.13)	− 0.30 (− 1.54, 0.94)
Other^d^	24,687	− 0.05 (− 0.20, 0.10)	− 0.21 (− 0.41, 0.004)	− 0.27 (− 0.49, − 0.05)	− 0.34 (− 0.58, − 0.11)
Comorbidities					
Heart and vascular	100,062	− 0.19 (− 0.32, − 0.05)	− 0.31 (− 0.47, − 0.16)	− 0.50 (− 0.62, − 0.37)	− 0.36 (− 0.53, − 0.20)
Cancer	39,368	0.14 (− 0.04, 0.31)	− 0.01 (− 0.20, 0.18)	− 0.18 (− 0.38, 0.01)	− 0.53 (− 0.84, − 0.23)
Lung	36,165	− 0.13 (− 0.30, 0.03)	− 0.12 (− 0.35, 0.10)	− 0.48 (− 0.76, − 0.20)	− 0.66 (− 1.12, − 0.20)
Renal	8949	− 0.01 (− 0.33, 0.32)	− 0.06 (− 0.45, 0.33)	− 0.47 (− 0.98, 0.04)	− 0.30 (− 1.46, 0.87)
Diabetes	24,416	− 0.36 (− 0.56, − 0.16)	− 0.49 (− 0.71, − 0.26)	− 0.84 (− 1.04, − 0.65)	− 0.89 (− 1.40, − 0.38)
Dementia	8100	− 0.37 (− 0.69, − 0.05)	− 0.50 (− 0.87, − 0.12)	− 0.46 (− 0.91, − 0.004)	0.32 (− 0.11, 0.74)
Immune	3140	− 0.22 (− 0.45, 0.02)	− 0.49 (− 0.972, − 0.003)	− 0.97 (− 1.71, − 0.21)	− 1.21 (− 2.21, − 0.21)
Liver	994	0.10 (− 0.79, 0.99)	− 0.46 (− 1.62, 0.69)	− 0.86 (− 2.15, 0.44)	− 1.79 (− 3.20, − 0.37)
All comorbidities^e^	154,382	− 0.19 (− 0.31, − 0.08)	− 0.30 (− 0.45, − 0.15)	− 0.46 (− 0.59, − 0.34)	− 0.47 (− 0.69, − 0.25)

*NA* not applicable, *ICU* intensive care unit

^a^Mortality adjusted according to the total age distribution in the total sample

^b^Period May 1, 2014, through December 31, 2021

^c^Infection side includes only those with implicit sepsis

^d^Other infections = Bone, obstetric, upper airway, central nervous system and unknown

^e^All comorbidities =  ≥ 1 comorbidity

Table 2 gives a detailed age-standardized percentage change per year in 30-day, 90-day, 1-, and 5-year mortality for implicit and explicit sepsis, sepsis patients admitted at ICU and wards, in addition to comorbidities and infection sites. Over time, sepsis patients had a decline in mortality, and patients with implicit sepsis had a larger decline in mortality than explicit sepsis patients. Further, ward patients had a larger decline than patients admitted to ICU, but this reversed at 1-year. Lastly, from 1-year after admission, the age-standardized mortality among all infection sites declined year by year, with largest decline in respiratory tract infections.

### Crude and age-standarized mortality

The median follow-up time in the study was 3.3 years (range 0 to 14 years). 30- and 60-day, 1- and 5-year crude and age-standardized mortality for all sepsis patients, and subgroups of implicit, explicit, and COVID-19-related sepsis patients, and also divided in sepsis patients admitted at intensive care or wards are shown in Table 3. Overall, sepsis patients had a 30-day age-standardized mortality of 16.9% (95% CI 16.7 to 17.0). COVID-19-related sepsis had the highest age-standardized 30-day mortality (21.5%; 95% CI 19.4 to 23.6) versus 15.9% (95% CI 15.7 to 16.1) for implicit sepsis and 18.5% (95% CI 18.2 to 18.7) for explicit sepsis. ICU patients had higher mortality than ward patients until one years after first sepsis admission, whereas the 5-year mortality was largely similar in ICU and ward patients.

**Table 3:** Crude and age-standardized mortality^a^ (%) at 30-day, 90-day, 1- and 5-year after first admission in different subgroups (2008–2021)

	Mortality							
	30-day (%)		90-day (%)		1-year (%)		5-year (%)	
	Crude	Adjusted^a^ (95% CI)	Crude	Adjusted^a^ (95% CI)	Crude	Adjusted (95% CI)	Crude	Adjusted^a^ (95% CI)
All sepsis patients^b^	16.9	16.9 (16.7, 17.0)	23.9	23.9 (23.7, 24.1)	34.3	34.3 (34.1, 34.5)	58.5	58.5 (58.2, 58.7)
Subgroup								
Implicit	16.6	15.9 (15.7, 16.1)	23.8	22.8 (22.6, 23.1)	34.5	33.2 (32.9, 33.4)	62.1	59.4 (59.1, 59.7)
Explicit	17.3	18.5 (18.2, 18.7)	24.3	25.7 (25.4, 26.0)	34.2	36.0 (35.7, 36.3)	54.5	57.4 (57.1, 57.8)
COVID-19-related sepsis^c^	13.1	21.5 (19.4, 23.6)	14.6	25.1 (22.5, 27.7)	20.4	27.7 (24.1, 31.5)	NA	NA
ICU patients^d^	22.7	26.0 (25.1, 26.9)	28.5	32.2 (31.2, 33.2)	36.1	40.9 (39.8, 41.9)	54.2	61.1 (59.6, 62.6)
Ward patients	16.6	16.5 (16.3, 16.6)	23.7	23.5 (23.3, 23.7)	34.2	34.0 (33.8, 34.2)	58.6	58.4 (58.2, 58.6)

*NA* not applicable, *ICU* intensive care unit

^a^Mortality adjusted according to the total age distribution in the total sample

^b^Crude and adjusted proportions are similar since the total study sample is used as the reference population

^c^Period from February 27, 2020, through December 31, 2021

^d^Period May, 1 2014, through December, 31 2021

### Characteristics associated with mortality

Compared to implicit sepsis patients, patients with explicit sepsis (HR 0.98; 95% CI 0.969 to 0.991) and COVID-19-related sepsis (adjusted HR 0.916; 95% CI 0.836 to 1.003) had similar risk of mortality. In the sensitivity analysis restricted to entry dates from February 27, 2020, we found a HR of 1.09 (95% CI 1.04 to 1.14) in patients with explicit sepsis and an adjusted HR of 0.85 (95% CI 0.77 to 0.93) in COVID-19-related sepsis patients, compared to patients with implicit sepsis (Supplementary Files, Supplementary Table 1).

Sepsis patients with respiratory tract infections had higher risk of dying compared to sepsis patients with other infections. Sepsis patients with cancer (adjusted HR 2.48; 95% CI 2.42 to 2.53), chronic lung disease (adjusted HR 1.21; 95% CI 1.18 to 1.24), dementia (adjusted HR 1.58; 95% CI 1.52 to 1.65), and chronic liver disease (adjusted HR 3.44; 95% CI 3.09 to 3.83) had higher risk of dying compared to the reference group with chronic vascular disease. Compared to sepsis patients with none comorbidities, sepsis patients with one, two and, three or more comorbidities had increasing adjusted HRs of 1.71 (95% CI 1.69 to 1.71), 2.12 (95% CI 2.09 to 2.16), and 2.60 (95% CI 2.54 to 2.67). Compared to sepsis patients with acute respiratory organ dysfunction, the adjusted HRs of long-term mortality was 1.05 (95% CI 1.02 to 1.08) for sepsis patients with acute circulatory dysfunction, 1.33 (95% CI 1.27 to 1.38) for sepsis patients with acute coagulation dysfunction, and 1.95 (95% CI 1.82 to 2.07) for sepsis patients with acute hepatic acute dysfunction. Further, having ≥ 2 acute organ dysfunctions was associated with higher long-term mortality than ≤ 1 acute organ dysfunction, adjusted HR 1.46 (95% CI 1.43 to 1.49), adjusted HR 2.02 (95% CI 1.93 to 2.11), and adjusted HR 3.04 (95% CI 2.78 to 3.32) for 2, 3 and ≥ 4 acute organ dysfunctions, respectively (Table 4).

**Table 4:** Hazard ratio for death from Cox regression by sepsis characteristics during follow-up of sepsis patients

Variable	No. of patients	Person year at risk	Deaths	Mortality per 100 person year	Crude HR	Adjusted HR^a^ (95% CI)
Sepsis subgroup						
Implicit	127,059	370,431	76,498	20.7	1.00	1.000 (Reference)
Explicit	92,928	356,820	54,738	15.3	0.86	0.980 (0.969–0.991)
COVID-19-related^b^	2845	1841	490	26.6	0.51	0.916 (0.836–1.003)
Site of infection^c^						
Respiratory	68,920	190,102	43,711	23.0	1.00	1.00 (Reference)
Genitourinary	27,311	87,844	16,416	18.7	0.83	0.68 (0.67–0.69)
Other infections^d^	24,450	85,671	12,610	14.7	0.70	0.84 (0.83–0.86)
Intra-abdominal	8857	27,536	5206	18.9	0.88	0.86 (0.84–0.89)
Gastrointestinal infections	8617	37,871	3844	10.2	0.52	0.58 (0.56–0.60)
Skin and soft tissue	5169	20,173	2395	11.9	0.58	0.65 (0.62–0.68)
Infections following a procedure	4111	18,082	1907	10.5	0.54	0.63 (0.61–0.66)
Endocarditis/myocarditis	1274	4186	731	17.5	0.83	1.01 (0.94–1.09)
Comorbidities^c^						
Heart and vascular	51,333	162,687	32,720	20.1	1.00	1.00 (Reference)
Cancer	21,614	45,614	16,272	35.7	1.52	2.48 (2.43–2.53)
Lung	14,062	47,214	8426	17.8	0.88	1.21 (1.18–1.24)
Diabetes	5434	23,163	2288	9.9	0.53	0.77 (0.74–0.81)
Dementia	2955	4578	2537	55.4	1.97	1.58 (1.52–1.65)
Renal	1902	4522	1055	23.3	0.96	1.01 (0.95–1.07)
Immune	1040	5230	341	65.2	0.37	0.91 (0.81–1.01)
Liver	463	955	335	35.1	1.55	3.44 (3.09–3.83)
No. of comorbidities						
0	68,450	305,693	25,516	8.3	1.00	1.00 (Reference)
1	98,803	293,964	63,974	21.8	2.28	1.71 (1.69–1.74)
2	45,352	109,290	33,963	31.1	3.00	2.12 (2.09–2.16)
≥ 3	10,227	20,145	8273	41.1	3.56	2.60 (2.54–2.67)
Type of acute organ dysfunction^c^						
Respiratory	49,234	139,667	30,855	22.1	1.00	1.00 (Reference)
Renal	53,010	154,416	30,879	20.0	0.90	0.68 (0.67–0.70)
Other acute organ dysfunctions^e^	17,954	67,926	9642	14.2	0.69	0.52 (0.51–0.53)
Circulatory	7425	18,784	4520	24.1	1.09	1.05 (1.02–1.08)
Coagulation	4820	14,881	2784	18.7	0.87	1.33 (1.27–1.38)
Hepatic	1365	2857	988	34.6	1.46	1.95 (1.82–2.07)
No. of acute organ dysfunctions						
1	133,808	398,531	79,668	20.0	1.00	1.00 (Reference)
2	15,262	36,114	9928	27.5	1.32	1.46 (1.43–1.49)
3	2864	6205	1881	30.3	1.48	2.02 (1.93–2.11)
≥ 4	699	1215	494	40.6	1.88	3.04 (2.78–3.32)
ICU treatment^f^						
No	114,423	261,062	56,398	21.6	1.00	1.00 (Reference)
Yes	10,602	23,034	5021	21.8	1.02	1.41 (1.37–1.46)

*HR* hazard ratio, *CI* confidence interval, *ICU*  intensive care unit

^a^Cox regression with time to death as dependent variable, the listed variable as covariate (one at the time), adjusted for per year 2009–2019 as continuous covariate, indicator covariates for the years 2008, 2020 and 2021, and sex and age

^b^Enter date = February 27, 2020

^c^Categorical variable where one ICD-10 code excludes other ICD-10 codes in the same diagnosis group

^d^Other infections = Bone, obstetric, upper airway, central nervous system and unknown

^e^Other acute organ dysfunctions = Acidosis, unspecific gangrene, central nervous system dysfunctions and Systemic Inflammatory Response Syndrome.

^f^Enter date = May 1, 2014

Patients treated in ICU had higher risk of death (adjusted HR 1.41; 95% CI 1.37 to 1.46) than those admitted to a general ward. Sensitivity analysis with binary categories is presented in Supplementary Files, Supplementary Table 2. In short, the sensitivity analysis showed that sepsis patients with respiratory infection had the highest risk of mortality (HR 1.38, 95% CI 1.27 to 1.30) compared to sepsis patients with other infection sites. Sepsis patients with cancer had the comorbidity with highest risk (HR 2.41, 95% CI 2.38 to 2.44) compared to sepsis patients with other comorbidities. Sepsis patients with acute hepatic organ dysfunction had the highest risk (HR 2.63 (95% CI 2.52 to 2.74) compared with sepsis patients with other organ dysfunctions.

(Supplementary Files, Supplementary results, Supplementary Table 3).

## Discussion

Our nationwide study is the first to provide contemporary estimate of mortality among sepsis patients over a 14-year period, including the recent pandemic, and in one joint paper include sepsis patients admitted to the general wards as well as ICU. Our study shows improvements in 30-day, 90-day, 1- and 5-year mortality from 2008 through 2021, with the largest decline among patients with sepsis due to respiratory tract infections. Moreover, we observe that long-term mortality varies according to the various infection sites, comorbidities, and acute organ dysfunction in patients admitted with a first sepsis episode. Lastly, it seems that COVID-19-related sepsis patients have largely the same mortality as explicit and implicit sepsis patients.

Previously, Rhee et al. (2017) compared clinical and claims data from the USA and found an in-hospital decline in mortality for explicit sepsis codes from 2009 to 2014^33^. Our findings are consistent with their study, but direct comparison of mortality reduction is challenging due to the various coding practices of sepsis. Additionally, two well-conducted meta-analyses of mortality trends in severe sepsis and septic shock patients using clinical trial data found a decline in mortality rates over time [34, 35]. The meta-analysis by Stevenssons and colleagues (2014) found an annual decrease of 3.0% in 28-day mortality [34], while the meta-analysis by Luhr et al. (2019) found an annual decrease of 0.42% in 28-day mortality, which was more pronounced in studies with a mean age ≥ 65 years. Our approach of using administrative databases to calculate mortality trends in sepsis patients is common [2, 5, 6, 36, 37], but not without controversy [10, 38]. The decline in mortality rates are often attributed to the Will-Rogers phenomenon, which explains reduced mortality as a consequence of including a larger proportion of less severely ill sepsis patients due to increased sepsis awareness [39]. However, in a recent study, we report an overall incidence of 246 per 100 000 person years among patients with a first sepsis admission, and that the incidence was stable from 2008 to 2021 [7]. Stable sepsis incidence is less likely to be explained by increased coding of less severe sepsis and indicates that the reduced mortality is unlikely to be explained by the Will Rogers phenomenon. Although mortality estimates using administrative data are overestimated compared to clinical data [33], our results are in line with the two meta-analyses studying clinical trials [34, 35].

Three recent observational studies by Vesteinsdottir (2021), Stranberg (2020) and Buchman (2021 found stable mortality trends [8, 9, 40]. In comparison, we observed decreasing short- and long-term mortality trends in mortality among ward patients, whereas for ICU patients the trend in 1-year mortality was stable. However, since Buchman et al. included patients with explicit sepsis ≥ 65 years and persons with disabilities and end-stage renal disease, it is likely that the diverging result is due to a more severe ill sample with a worse prognosis. The discrepancy in the results compared to Vesteinsdottir (2021) and Stranberg (2020) may be due to underestimation of the number of sepsis patients. Vesteinsdottir et al. (2021) excluded patients who developed severe sepsis or septic shock while admitted to the ICU for another admission diagnosis [8], while Stranberg et al. (2020) used the Swedish Intensive Care Registry [9], which is reported to underestimate the incidence of sepsis [41]. Our study, in contrast, utilized a large and diverse population-based sample of all sepsis admissions in Norway, including patients developing sepsis while admitted, during a 14-year study period. As the majority of sepsis patients are treated in wards, comparing our study with previous studies limited to selected ICU cohorts is challenging; however, our study’s contribution to understanding sepsis mortality among all sepsis patients is important for health care resource planning.

Stressing the importance of identifying the site of infection in sepsis management could have increased awareness and therefore improved the efforts to determine the site of infection. Our study found that the short-term mortality among patients admitted with known infection site (implicit sepsis) was lower than those admitted with unknown infection site (explicit sepsis), but that this reversed with longer observation time. One possible explanation can be that more patients in the explicit group had zero comorbidities and thus supposedly better long-term outcomes than those with comorbidities [22]. Further, one previous study evaluated in-hospital mortality trends stratified by site of infection in sepsis patients. They found that mortality from all infection sites had decreased significantly, with the largest decrease in skin/skin structure, primary bacteremia, and catheter-related bloodstream infections [18]. The annual decrease was much higher than in the current study, and for comparison, we had a higher number of respiratory tract infections and a lower number of skin infections, in addition to a longer follow-up time. Further, the decline in mortality trends among patients with respiratory tract infections in our study can in some extent be explained by pneumococcus vaccinations [42] and the relatively low bacterial resistance in Norway [43].

The literature on the association between infection sites and mortality also provides conflicting results. A Danish study (2016) found that urinary tract infection was an independent predictor of mortality [44], while a long-term follow-up of ICU patients in England (2019) found that all infection sites had a lower adjusted hazard ratio compared to respiratory tract infections [4]. The latter study is consistent with our results. Another study by Nygård et al. (2013) identified endocarditis/myocarditis and intra-abdominal infections as independent predictors of poor outcomes [19]. These differences in results may be due to variations in follow-up, study design, and selection of cohorts.

Our study found a strong association between liver dysfunction and long-term mortality, which is in line with previous findings [25, 26]. Similarly, a study with three year of follow-up found acute liver dysfunction to be strongly associated with long-term mortality, together with acute coagulation and acute neurologic dysfunction in sepsis survivors [25]. However, the effect size of acute liver dysfunction in these studies was smaller than in ours, which can be explained by differences in study population (sepsis patients at ICU or sepsis patients who went through the emergency department versus all hospital departments), data sources to identify sepsis patients (SOFA-scores versus discharge codes) and inclusion criteria (sepsis patients surviving hospital stay versus all patients admitted with sepsis for the first time). Including only sepsis patients that survive discharge can cause an underestimation of the severity of sepsis, thus affecting the association between clinical characteristics and mortality. In the planning of our study, an expert panel found acute neurologic dysfunction codes to come with great uncertainty, especially among sepsis patients at high age. Therefore, acute neurological dysfunctions were not categorized as a single dysfunction, thus making comparisons for the number of organ dysfunctions and mortality risk challenging. Furthermore, we did not have the possibility to exclude end-stage comorbidity diseases, possibly contributing to a stronger association between acute organ dysfunctions and mortality.

To our knowledge, no previous study has investigated the long-term mortality of implicit, explicit, and COVID-19-related sepsis in one joint study. Interestingly, in light of all the media coverage directed toward the COVID-19-patients’ risk of death, the mortality in patients with COVID-19-related sepsis was similar to patients with implicit and explicit sepsis. In sensitivity analysis restricted to the pandemic years 2020 and 2021 the risk of death was slightly lower for COVID-19-related sepsis patients. We also found that the frequencies of underlying comorbid diseases among patients admitted with implicit, explicit and COVID-19-related sepsis in our study were higher compared to the previously reported prevalence of comorbidity in the general population in Norway [45]. These results emphasize the need to discuss the recourses used after discharge including all sepsis patients, not focused to COVID-19 patients. Further, we found that implicit sepsis patients had the same risk as explicit sepsis patients. This is in contrast to a nationwide study based on ICD-10 codes, and a study investigating mortality trends comparing clinical versus claims data, and found that explicit sepsis had a higher in-hospital mortality [33, 37]. For comparison, some of the diverging results can be explained by ICD-10 code selection and search strategies, where they included other combinations of ICD-10 codes to identify implicit sepsis and searched in a lower number of secondary diagnoses to combine infection and organ dysfunction. The latter can contribute to a underestimation of sepsis, especially implicit sepsis, and therefore recommended approach is to search in minimum 15 diagnosis fields to capture sepsis [46].

Surprisingly, only 8.5% of the sepsis patients received ICU treatment. In comparison, a recent French nationwide study found that over 50% of sepsis patients received ICU treatment [37]. Possible explanation of this diverging result can be that the ICU capacity in Norway is found to be in the lower range [47]. In addition, some of the less severe ill sepsis patients can be admitted at intermittent wards (not defined as ICUs) that manage acute organ dysfunctions, including non-invasive ventilation and medical treatment for low blood pressure..

## Strengths and limitations

This study has several strengths. We included 222,832 patients with a first hospitalization of sepsis from 2008 to the end of 2021 in all Norwegian hospitals, which enabled us to conduct reliable subgroup analysis and examine recent survival trends. NPR and The Norwegian Cause of Death Registry are both widely used in research and have minimal missing data [31, 48]. Reporting to all three registries used are mandatory and followed by yearly quality controls, which limits participation bias due to completeness. Using the Norwegian Patient Registry also allows us to avoid survivor bias, as we have the date of admission to hospital for all patients, not only those who survive hospital. Further, using the Norwegian cause of death registry enables us complete follow-up to death date, thus avoiding attrition bias. Further, also the variable ICU-admission (yes/no) is expected to be complete since weekly reporting to ensure sufficient health care planning was mandatory the first two pandemic years. This amplifies the correctness of the ICD-10 codes in our study period. Another strength is that we, in one study, report the overall age-standardized long-term mortality for implicit, explicit, and COVID-19-related sepsis. To the best of our knowledge, this is the first study that provides nationwide trends in long-term mortality in patients admitted with sepsis over 14 years with separate analyses for patients admitted at ICU and ward patients and includes COVID-19-related sepsis.

There are also several limitations to our study. First, the use of registry-based study design is dependent on ICD-code abstraction [38], and different extractions of ICD-codes have been investigated to find the most fitted design, with diverging results [49–52]. In global counting of sepsis, Rudd et al. (2020) has been criticized for code-selecting strategies, that one strategy do not fit all countries, and most probable cause an overestimation of sepsis [53]. Fleishmann-Struzek (2018) compared the validity of different ICD coding for sepsis in Germany and found that explicit sepsis coding had a positive predictive value (PPV) of 59.6% and a threefold risk of underestimating sepsis incidence, while implicit sepsis had a PPV of 22.1%, and a 2.7-fold risk overestimating sepsis incidence [54]. The systematic review by Jolley et al. (2015) concludes that sepsis is largely undercoded in administrative data using ICD-9 and ICD-10 codes [55]. Our approach, using both explicit and implicit sepsis codes, may be in line with the under- and overestimation of explicit and implicit sepsis coding strategies described in these above studies. The strategy was designed to capture ICD-10 codes used to identify sepsis in Norway and included search for both explicit and implicit codes in 20 secondary diagnosis fields, which is in line with recommandations [46]. The ICD-10 codes are not static, and new codes for SIRS and septic shock were implemented in 2010 [56]. We used the Sepsis-3 definition during the entire study period, albeit the new definition first came in 2016 [1]. Second, retrieving organ dysfunction codes to identify implicit sepsis can generate false-positive outcomes since not all organ dysfunctions are caused by a specific infection. On the other hand, false-negative results can occur if the sepsis episode is inadequately documented. Third, although we did separate analysis for patients receiving intensive care treatment, we cannot rule out the possibility that illness severity could have influenced the risk differences observed between subgroups of patients. Fourth, presenting results adjusting for age and sex could mask possible age or sex specific associations with mortality. Finally, the level of SARS-CoV-2 incidence in Norway has been relatively low, and therefore, it can be speculated that mortality after COVID-19-related sepsis would have been different if the capacity in hospitals and ICUs was exceeded, as reported from other countries [57, 58].

Our results have implications for health policymakers, clinicians, and researchers. Although the case fatality is decreasing, sepsis survivors have high mortality in months and years after discharge. Long-term mortality in sepsis survivors requires further attention as more sepsis survivors put more pressure on skilled nursing facilities and in-home care.

## Conclusion

This is the first study including sepsis patients admitted at wards and ICU that during fourteen years (2008–2021) demonstrates decreasing long-term mortality. Decrease was observed for all sepsis patients and all infections sites but was largest among patients with respiratory tract infections. Lastly, it seems that COVID-19-related sepsis patients have the same mortality risk as explicit and implicit sepsis patients.

### Supplementary Information

Below is the link to the electronic supplementary material.

Supplementary file1 (DOCX 93 KB)

## Data Availability

No additional data available.
