# Correction: Trends in mortality after a sepsis hospitalization: a nationwide prospective registry study from 2008 to 2021

**DOI:** 10.1007/s15010-023-02090-z

**Published:** 2023-09-21

**Authors:** Nina Vibeche Skei, Tom Ivar Lund Nilsen, Randi Marie Mohus, Hallie C. Prescott, Stian Lydersen, Erik Solligård, Jan Kristian Damås, Lise Tuset Gustad

**Affiliations:** 1 Department of Anesthesia and Intensive Care, Nord-Trondelag Hospital Trust, Levanger, Norway; 2 Department of Circulation and Medical Imaging, Mid Norway Sepsis Research Center, Norwegian University of Science and Technology (NTNU), Trondheim, Norway; 3 Department of Public Health and Nursing, Norwegian University of Science and Technology (NTNU), Trondheim, Norway; 4 Clinic of Anesthesia and Intensive Care, St. Olav’s University Hospital, Trondheim, Norway; 5 Department of Internal Medicine, University of Michigan, Ann Arbor, MI USA; 6 VA Center for Clinical Management Research, Ann Arbor, MI USA; 7 Department of Mental Health, Faculty of Medicine and Health Sciences, Regional Centre for Child and Youth Mental Health and Child Welfare, Norwegian University of Science and Technology, (NTNU), Trondheim, Norway; 8 Centre of Molecular Inflammation Research, Institute for Clinical and Molecular Medicine, Norwegian University of Science and Technology (NTNU), Trondheim, Norway; 9 Department of Infectious Diseases, St. Olav’s University Hospital, Trondheim, Norway; 10 Faculty of Nursing and Health Sciences, Nord University, Levanger, Norway; 11 Department of Medicine and Rehabilitation, Levanger Hospital, Nord-Trøndelag Hospital Trust, Levanger, Norway

**Correction: Infection** 10.1007/s15010-023-02082-z

Table 1 was displayed incorrectly. The Table 1 should appeared as shown below.

**Table 1:** Characteristic of the study population with sepsis (2008–2021), including subgroups

	Sepsis^a^	Subgroups of sepsis		
		Implicit^b^	Explicit^c^	COVID-19-related^d^
Characteristics				
First admission, *n* (% of all)	222,832 (100)	127,059 (57.0)	92,928 (41.7)	2845 (1.3)
Male, *n* (%)	120,442 (54.1)	66,929 (52.7)	51,651 (55.6)	1862 (65.5)
Mean age, years (SD)	71.1 (16.6)	73.0 (15.7)	68.9 (17.5)	61.4 (16.1)
Comorbidities, *n* (%)				
Heart and vascular	100,062 (44.9)	61,251 (48.2)	38,109 (41.0)	702 (24.7)
Cancer	39,368 (17.7)	17,270 (13.6)	21,973 (23.6)	125 (4.4)
Lung	36,165 (16.2)	26,993 (21.2)	8866 (9.5)	306 (10.8)
Renal	8949 (4.0)	5830 (4.6)	3043 (3.3)	76 (2.7)
Diabetes	24,416 (10.9)	13,682 (10.8)	10,348 (11.1)	386 (13.6)
Dementia	8100 (3.6)	4561 (3.6)	3507 (3.8)	32 (1.1)
Immune	3140 (1.4)	1640 (1.3)	1451 (1 0.6)	49 (1.7)
Liver	994 (0.5)	564 (0.4)	427 (0.5)	≤ 5
Number of comorbidities, *n* (%)				
0	68,450 (30.7)	36,185 (28.5)	30,684 (33.0)	1581(55.6)
1	98,803 (44.3)	56,884 (44.7)	41,050 (44.2)	909 (32.0)
2	45,352 (20.4)	27,768 (21.9)	17,284 (18.6)	300 (10.5)
≥ 3	10,227 (4.6)	6262 (4.9)	3910 (4.2)	55 (1.9)
Site of infection, *n* (%)				
Respiratory	81,881 (36.8)	63,724 (50.2)	15,566 (16.8)	2591 (91.1)
Genitourinary	44,782 (20.1)	28,838 (22.7)	15,862 (17.1)	82 (2.9)
Skin and soft tissue	8265 (3.7)	3578 (2.8)	4682 (5.0)	5 (0.2)
Gastrointestinal	10,810 (4.8)	8356 (6.6)	2424 (2.6)	30 (1.1)
Intra-abdominal	12,340 (5.5)	5401 (4.3)	6917 (7.4)	22 (0.8)
Infections following a procedure	8290 (3.7)	4042 (3.2)	4235 (4.6)	13 (0.5)
Endocarditis/myocarditis	2530 (1.1)	1008 (0.8)	1514 (1.6)	8 (0.3)
Other^e^	43,085 (19.3)	24,463 (19.3)	18,434 (19.8)	188 (6.6)
Organ system with acute dysfunction, *n* (%)				
Respiratory	61,864 (27.8)	51,453 (40.5)	8012 (8.6)	2399 (84.3)
Circulatory	14,892 (6.7)	10,647 (8.4)	4177 (4.5)	68 (2.4)
Renal	67,242 (30.2)	54,295 (42.7)	12,514 (13.5)	433 (15.2)
Hepatic	3209 (1.4)	2178 (1.7)	1014 (1.1)	17 (0.6)
Coagulation	6471(2.9)	3858 (3.1)	2570 (2.8)	43 (1.5)
Other^f^	22,173 (10.0)	20,095 (15.8)	1928 (2.1)	150 (5.3)
Number of acute organ dysfunctions, *n* (%)				
1	133,808 (87.7)	113,998 (89.7)	17,339 (76.0)	2471 (86.9)
2	15,262 (10.0)	11,038 (8.7)	3955 (17.3)	269 (9.5)
3	2864 (1.9)	1693 (1.3)	1144 (5.0)	27 (0.9)
≥ 4	699 (0.5)	330 (0.3)	264 (1.6)	≤ 5
Number of hospital admissions for sepsis^g^, *n* (%)				
1	171,619 (77.0)	97,105 (76.4)	71,800 (77.3)	2714 (95.4)
2	33,221 (14.9)	19,339 (15.2)	13,757 (14.8)	125 (4.4)
3	10,129 (4.6)	5917 (4.7)	4208 (4.5)	≤ 5
4	4011 (1.8)	2363 (1.9)	1647 (1.8)	≤ 5
≥ 5	3852 (1.7)	2335 (1.8)	1516 (1.6)	≤ 5
Readmission^h^, *n* (%)	55,441 (24.9)	30,895 (24.3)	24,072 (25.9)	474 (16.7)
ICU treatment^j^, *n* (%)	10,602 (8.5)	6946 (8.7)	3341 (7.9)	315 (11.1)
In-hospital death, *n* (%)	30,276 (13.6)	16,273 (12.8)	13,751 (14.8)	352 (12.4)

*ICU* intensive care unit

^a^Sepsis = All first sepsis admissions in the period 2008–2021, including implicit, explicit and COVID-19-related sepsis (2020–2021)

^b^Implicit sepsis = ICD-10 code for infection in combination with a code for acute organ function, excluding those who had an explicit code at the same hospital admission

^c^Explicit sepsis = ICD-10 code for specific sepsis, including those who also had an implicit code at the same admission

^d^COVID-19-related sepsis = ICD-10 code for COVID-19 in combination with an acute organ dysfunction code and/or a specific sepsis code

^e^Other infections = Bone, obstetric, upper airway, central nervous system and unknown

^f^Other acute organ dysfunction = Acidosis, unspecific gangrene, central nervous system dysfunctions and Systemic Inflammatory Response Syndrome

^g^Number of hospital admissions = Calculated as new sepsis admission if admission with ICD-10 codes defining sepsis, regardless of time frame for the new sepsis admission

^h^Readmission = admission within 30 days after discharge regardless of cause

^j^Variable calculated from May 1, 2014

The original article has been corrected.

