# Supplementary material for: Trends in mortality after a sepsis hospitalization: a nationwide prospective registry study from 2008 to 2021
Source: Infection. 2023 Aug 12;51(6):1773–86. doi: 10.1007/s15010-023-02082-z (PMC10665235; doi:10.1007/s15010-023-02082-z)
Supplement: Supplementary file 1 — Supplementary file1 (DOCX 93 KB) [file 15010_2023_2082_MOESM1_ESM.docx]

Supplementary Files

**Supplementary Methods**

We used ICD-10 codes from the Norwegian Patient Registry (NPR) to define sepsis as implicit (known infection plus organ dysfunction), explicit (unknown infection) and COVID-19 related-sepsis

*Implicit sepsis* was retrieved with ICD-10 codes for infection together with ICD-10 codes for acute organ dysfunction in the same hospital admission. Here is a list of the codes we combined for infection and acute organ dysfunction:

ICD-10 codes for infection:

A00/09, A19/28, A30/32, A36/39, A42/ 44, A46, A48/49, A54, A59, A69.0, A69.1, A69.9, A70, A74/75, A77/81, A83/89, A92/99, B00/09, B25/27, B33/34, B37/46, B48/50, B54/55, B57/58, B60, B64, B67, B95/97, B99, G00/08, H05.0, H60.2, H70.0, I00, I33, I38/40.0, J01/06, J09/22, J36, J39.0, J39.1, J85, J86, K35/37, K61, K63.0/63.1, K65, K75.0, K81.0, K83.0, L02/04, L08, M00/01, M86, M72.6,N10, N15.1, N30, N39.0, N41.0, N41.2, N41.3, N45, N70/74, N98.0, N49, O03.0, O03.5, O04.5, O08.0, O23, O75.3, O85/86, O88.3, O91, O98, T80.2, T81.4, T82.6/82.7, T83.5/83.6, T84.5/84.7, T85.7, T88.0, U04

ICD-10 codes for acute organ dysfunction

D65, D69.5, E87.2, G93.4, I46, I95.9, J80, J95.2, J96, K72.0, K72.9, N00, N17, N99.0, R02, R09.0, R09.2, R40.0/40.2, R41, R55, R57, R57.2, R65.1

*Explicit sepsis* was retrieved for these ICD-10 codes:

A02.1, A20.7, A21.7, A22.7, A24.1, A26.7, A28.2, A32.7, A39.2, A39.4, A40, A41, A42.7, B00.7, B37.7

*COVID-19-related sepsis* was retrieved using ICD-10 codes for COVID-19-infection U07.1 and U07.2 together with codes for acute organ dysfunction. We used the same codes for acute organ dysfunction as for implicit sepsis (see above).

*Comorbidities* was also retrieved using ICD-10 codes. In the selection process we defined comorbidities from groups of comorbidities. Here is a list of how we categorized the comorbidities according to ICD-10-codes::

**Chronic heart- and vascular disease**; G45, H34, I00/31, I34/37, I42/45, I47/95.8, I97/99. **Cancer**; C00/97, D32/33, D35.2/35.4, D42, D43, D44.3/44.5, D45/47. **Chronic lung disease**; J41/47, J84, J98. **Chronic renal disease**; N18.3/18.5. **Diabetes;** E10/11. **Dementia**; F00/03, G30, G31.0, G31.2, G31.8. **Chronic immune disease**; D80/84, Z94.0/94.4, Z94.8. **Chronic liver disease**; K70.4, K72.

*Infection Site* was categorized according to these ICD-10 diagnoses: :

**Respiratory**; J09/18, J20/22, J85/86, U04. **Genitourinary**: N10, N15.1, N30, N39.0, N41.0, N41.2/41.3, N45, N49, N70, N71/74, N98.0; **Gastrointestinal-infections**; A00/09. **Intra-abdominal**; K35/37, K57, K61/61.1 K61.3, K63.0/63.1, K65, K75.0, K81.0, K83.0. **Endocarditis/myocarditis**; I32/33, I38/41. **Skin/ Soft tissue**; A46, B08/09, L02/04, L08, M72.6. **Infection after procedure**; T80.2, T81.4, T82.6/82.7, T83.5/83.6, T84.5/84.7, T85.7, T88. **Other**; A19/28, A30/32, A36/39, A42/44, A48/49, A54, A59, A69.0, A69.1, A69.9, A70, A74/75, A77/80, A81, A83/89, A92/B06, B25/27, B33/34, B37/46, B48/50, B54/55, B57/58, B60, B64, B67, B95/97, B99, G00/08, H05.0, H60.2, H70.0, J01/06, J36, J39.0/39.1, M00/01, M86, O03.0, O03.5, O04.5, O08.0, O23, O75.3, O85/86, O88.3, O91, O98.

*Acute organ dysfunction* was categorized according to these ICD-10 diagnoses::

**Respiratory**; J80, J95.2, J96, R09.0, R09.2. **Circulatory**; I46, I95.9, R57, R57.2. **Renal**; N00, N17, N99.0. Hepatic; K72.0, K72.9. **Coagulation**; D65, D69.5. **Other acute organ dysfunctions**; G93.4, R40.0/40.2, R41, R55, E87.2, R02, R65.1.

**Supplementary Results**


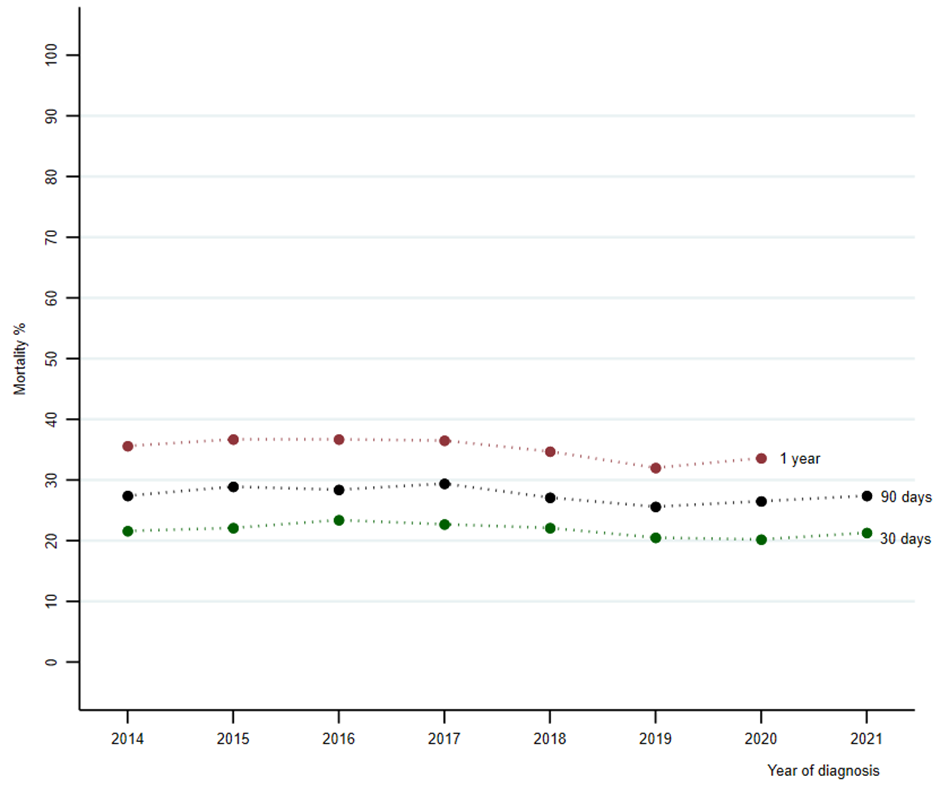
**Supplementary Figure 1** Age-standardized mortality at 30- and 90-day and 1-year for patients with sepsis receiving intensive care treatment

| **Supplementary Table 1** Hazard ratio for death from Cox regression by sepsis characteristics during follow-up of sepsis patients. | | | | | | | |
| --- | --- | --- | --- | --- | --- | --- | --- |
| Variable | No. of patients | Person year at risk | Deaths | Mortality  per 100 person year | Crude HR | Adjusted HR^a^ (95% CI) | |
| Sepsis subgroup^b^ |  |  |  |  |  |  |  |
| Implicit | 17 315 | 11 079 | 5 598 | 50.5 | 1.00 | 1.00 (Reference) |  |
| Explicit | 8 258 | 5 526 | 2 660 | 48.1 | 0.99 | 1.09 (1.04–1.14) |  |
| COVID-19-related | 2 845 | 1 841 | 490 | 26.6 | 0.53 | 0.85 (0.77–0.93) |  |
| Abbrevation: HR=Hazard Ratio, CI= Confidence Interval.  ^a^ Cox regression with time to death as dependent variable, the listed variable as covariate, adjusted for indicator covariate for the year 2020, sex and age.  ^b^ All patients entered after 27^th^ February 2020 | | | | | | |  |

| **Supplementary Table 2** Hazard ratio for death from Cox regression by sepsis characteristics during follow-up of sepsis patients. | | | | | | |
| --- | --- | --- | --- | --- | --- | --- |
| Variable | No. of patients | Person year at risk | Deaths | Mortality  per 100 person year | Crude HR | Adjusted HR^a^ (95% CI) |
| Infection site |  |  |  |  |  |  |
| Respiratory^b^ | 81 881 | 225 907 | 51 954 | 23.0 | 1.31 | 1.28 (1.27–1.30) |
| Genitourinary^b^ | 44 782 | 128 256 | 25 781 | 18.6 | 0.95 | 0.76 (0.75–0.77) |
| Intra-abdominal^b^ | 12 340 | 39 111 | 6 995 | 17.9 | 0.99 | 0.99 (0.97–1.02) |
| Gastrointestinal infections^b^ | 10 810 | 44 706 | 5 126 | 11.5 | 0.67 | 0.72 (0.70–0.74) |
| Skin and soft tissue^b^ | 8 265 | 20 173 | 2 395 | 11.9 | 0.69 | 0.80 (0.78–0.82) |
| Infections following a procedure^b^ | 8 290 | 33 392 | 3 674 | 11.0 | 0.64 | 0.74 (0.72–0.77) |
| Endocarditis/myocarditis^b^ | 2 530 | 8 033 | 1 371 | 17.1 | 0.94 | 1.09 (1.03–1.15) |
| Other infections^b,c^ | 43 085 | 141 556 | 21 391 | 15.1 | 0.75 | 0.86 (0.85–0.87) |
| Comorbidities |  |  |  |  |  |  |
| Heart and vascular^d^ | 100 062 | 277 421 | 69 884 | 25.2 | 1.66 | 1.14 (1.13–1.16) |
| Cancer^d^ | 39 368 | 75 212 | 30 876 | 41.1 | 2.10 | 2.41 (2.38–2.44) |
| Lung^d^ | 36 165 | 100 190 | 25 675 | 25.6 | 1.37 | 1.32 (1.31–1.43) |
| Diabetes^d^ | 24 416 | 78 015 | 15 519 | 19.9 | 1.08 | 1.03 (1.01–1.05) |
| Dementia^d^ | 8 100 | 11 800 | 7 187 | 60.9 | 2.39 | 1.47 (1.43–1.51) |
| Renal^d^ | 8 949 | 17 553 | 6 140 | 35.0 | 1.50 | 1.24 (1.21–1.27) |
| Immune^d^ | 3 140 | 12 369 | 1 535 | 12.4 | 0.72 | 1.21(1.16–1.28) |
| Liver^d^ | 994 | 1 902 | 746 | 39.2 | 1.83 | 3.01 (2.81–3.25) |
| Type of acute organ dysfunction |  |  |  |  |  |  |
| Respiratory^e^ | 61 864 | 169 225 | 39 335 | 23.2 | 1.29 | 1.47 (1.45–1.48) |
| Renal^e^ | 67 242 | 186 433 | 40 216 | 21.6 | 1.15 | 0.95 (0.94–0.96) |
| Circulatory^e^ | 14 982 | 35 239 | 9 278 | 26.3 | 1.38 | 1.55 (1.52–1.59) |
| Coagulation^e^ | 6 471 | 18 820 | 3 831 | 20.4 | 1.08 | 1.72 (1.67–1.78) |
| Hepatic^e^ | 3 209 | 6 232 | 2 337 | 37.5 | 1.84 | 2.63 (2.52–2.74) |
| Other acute organ dysfunctions^e,f^ | 22 173 | 78 441 | 12 249 | 15.6 | 0.85 | 0.72 (0.70–0.73) |
| Abbrevation: HR=Hazard Ratio, CI= Confidence Interval.  ^a^ Cox regression with time to death as dependent variable, the listed variable as covariate (one at the time), adjusted for per year 2009-2019 as continuous covariate, indicator covariates for the years 2008, 2020 and 2021, and sex and age.  ^b^ Reference group= All other infection sites  ^c^ Other infections= Bone, obstetric, upper airway, central nervous system and unknown  ^d^ Reference group= All other comorbidities  ^e^ Reference group= All other acute organ dysfunctions  ^f^ Other acute organ dysfunctions= Acidosis, unspecific gangrene, central nervous system dysfunctions and Systemic Inflammatory Response Syndrome. | | | | | | |

| Supplementary Table 3 Hazard ratio for death from Cox regression by admission year 2008-2021 of sepsis patients. | | |
| --- | --- | --- |
|  | Crude HR | Adjusted^a^ HR ( 95% CI) |
| 2008 | 1.059 | 1.058 (1.037-1.080) |
| 2009 | 1 | 1 |
| 2010 | 0.984 | 0.975 (0.973–0.976) |
| 2011 | 0.969 | 0.950 (0.946–0.953) |
| 2012 | 0.954 | 0.930 (0.920–0.931) |
| 2013 | 0.934 | 0.902 (0.895–0.909) |
| 2014 | 0.925 | 0.879 (0.870–0.888) |
| 2015 | 0.910 | 0.857 (0.846–0.867) |
| 2016 | 0.896 | 0.835 (0.823–0.846) |
| 2017 | 0.882 | 0.813 (0.801–0.826) |
| 2018 | 0.869 | 0.793 (0.779–0.807) |
| 2019 | 0.855 | 0.773 (0.757–0.788) |
| 2020 | 0.884 | 0.794 (0.772–0.817) |
| 2021 | 0.904 | 0.829 (0.803–0.856) |
| ^a^ Cox regression with time to death by year as dependent variable, unadjusted (crude), and adjusted for age and sex. The year 2009 is reference. | | |
